# Enhanced Surface Properties of Light-Trapping Si Nanowires Using Synergetic Effects of Metal-Assisted and Anisotropic Chemical Etchings

**DOI:** 10.1038/s41598-019-52382-4

**Published:** 2019-11-04

**Authors:** Youngsoon Jeong, Chanwoo Hong, Yeong Hun Jung, Rashida Akter, Hana Yoon, Ilsun Yoon

**Affiliations:** 1 Department of Chemistry, Chungnam National University, Daejeon, 34134 Republic of Korea; 2 Korea Institute of Energy Research (KIER), Daejeon, 34129 Republic of Korea

**Keywords:** Nanowires, Solar cells

## Abstract

Metal-assisted chemical etching (MACE) has been widely explored for developing silicon (Si)-based energy and optical devices with its benefits for low-cost and large-area fabrication of Si nanostructures of high aspect ratios. Surface structures and properties of Si nanostructures fabricated through MACE are significantly affected by experimental and environmental conditions of etchings. Herein, we showed that surfaces and interfacial energy states of fabricated Si nanowires can be critically affected by oxidants of MACE etching solutions. Surfaces of fabricated Si nanowires are porous and their tips are fully covered with lots of Si nano-sized grains. Strongly increased photoluminescence (PL) intensities, compared to that of the crystalline Si substrate, are observed for MACE-fabricated Si nanowires due to interfacial energy states of Si and SiO_x_ of Si nano-sized grains. These Si grains can be completely removed from the nanowires by an additional etching process of the anisotropic chemical etching (ACE) of Si to taper the nanowires and enhance light trapping of the nanowires. Compared with the MACE-fabricated Si nanowires, ACE-fabricated tapered Si nanowires have similar Raman and PL spectra to those of the crystalline Si substrate, indicating the successful removal of Si grains from the nanowire surfaces by the ACE process.

## Introduction

Silicon (Si) nanowires have been extensively studied due to their unique properties and great potentials in applications for optical and electrical sensors and energy conversion and storage devices^1–4^. Simple and large-area fabrications of vertically aligned Si nanowires have been widely developed for efficient and cost-effective Si photovoltaics due to their benefits of efficient light trapping and short carrier collection paths^5–7^. Metal-assisted chemical etching (MACE), in which Si is vertically etched in a mixed solution of HF and H_2_O_2_ with a patterned catalyst of noble metals (e.g., Ag and Au), has attracted significant interest as a simple and cost-effective etching method for fabricating vertically aligned Si nanowires^8–10^.

**Figure 1:**
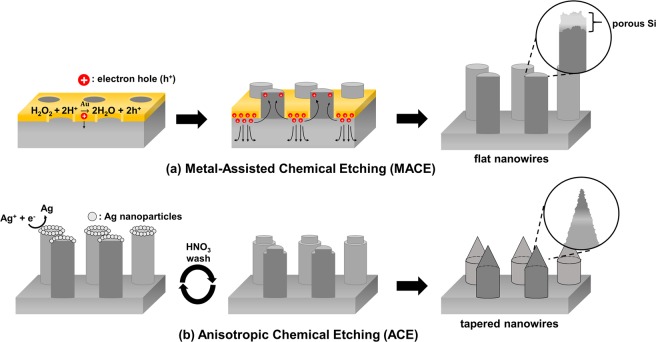
Schematic of the fabrication processes of vertically etched flat and tapered Si nanowires. (**a**) Si nanowires are vertically etched by MACE of a Si (100) substrate. The holes, generated excessively on the interface of the Au mask and the Si substrate during the MACE process, can be diffused and accumulated in the Si substrate and Si nanowires. These accumulated holes can make the nanowire surfaces oxidized and porous with Si nano-sized grains during and after the MACE process. (**b**) Unstable Si atoms and Si grains can be effectively removed from the nanowire surface by performing multiple rounds of ACE, which etches the nanowires selectively to obtain tapered shapes.

MACE is based on simple redox reactions those occur on the interfaces of catalytic metals and a Si substrate^1,2,11–13^. Holes (h^+^), created by the reduction of H_2_O_2_ on the metal surface, are transferred to the underlying Si substrate and oxidize the Si atoms of the substrate as schemed in Fig. 1(a)^2,11–13^. These oxidized Si atoms are then etched by HF in the etching solution. The underlying Si substrate can be vertically etched as MACE of the Si substrate is proceeded continuously.

In the fabrication of vertically aligned Si nanowires through MACE, the length of nanowires are usually controlled with the reaction time and the diameter and interspace of the nanowires are controlled with the patterned structure of catalytic metals, respectively^14,15^. The reaction rates of MACE and the structures of Si nanowires can be critically affected by the reaction conditions including materials and surface structures of catalytic metals, reaction temperatures, compositions of the etching solution, crystallographic orientations, and types and concentrations of dopants of the Si substrate^1,11,16,17^. In MACE, the holes transferred to the Si substrate are mainly used up for the oxidization of Si atoms near the catalytic metals^10,15,18,19^. However, these holes can result in additional oxidations and etchings of the surface and interior of the Si nanowires and substrates if excess holes are created on the catalytic metals and diffused into the Si substrate and nanowires^2,11,16^. Since the oxidant concentration in the etching solution is critical for hole generations on the catalytic metal surface, surface structures and morphologies of fabricated Si nanowires can be significantly affected by the concentration of H_2_O_2_ in the etching solution^1,13,20^. By this reason, the concentration of H_2_O_2_ needs to be optimized for fabricating Si nanowires through MACE.

In this study, we investigate the influences of H_2_O_2_ on Si nanowire surfaces and on their interfacial energy states during the MACE process with electron microscopy, and Raman and photoluminescence (PL) spectroscopy studies. Surfaces of MACE-fabricated Si nanowires are highly porous and their tips are covered with lots of Si nano-sized grains which could be easily oxidized during and after the MACE process. The formation and oxidization of the Si nano-sized grains on the nanowire surfaces are strongly affected by etching procedures and conditions^8,18,20–22^, contributing in significant increases in PL intensities of the nanowires^9,17^. These Si nano-sized grains and their interfaces can be completely removed from the nanowire surface by selective etching of reactive Si atoms with the anisotropic chemical etching (ACE). The Si nanowires are vertically tapered during the ACE processes, to afford sharp tip ends, significantly enhanced light trapping and highly suppressed PL emission^5,6,8,21–23^. To better understand how surface oxidation of the Si nanowires during the MACE process will affect on crystallinities and interfacial energy states of the tapered Si nanowires of the ACE process, which is the post-etching process of the MACE process, changes in surface morphologies and optical properties of the tapered nanowires are compared with those of the MACE fabricated nanowires.

## Results and Discussion

The MACE mechanism is generally considered to involve several redox reactions near the catalytic metal surface including H_2_O_2_ reduction in the etching solution on the catalyst surface (Eq. 1), injection of holes into the underlying Si substrate, oxidation of Si atoms due to the injected holes (Eq. 2) and etching of the oxidized Si atoms by HF^1,11^. The half reactions of reduction and oxidation involved in MACE with Au catalysts can be described as follows^9,23,24^:

1 $${\bf{C}}{\bf{a}}{\bf{t}}{\bf{h}}{\bf{o}}{\bf{d}}{\bf{e}}:{{\rm{H}}}_{{\rm{2}}}{{\rm{O}}}_{{\rm{2}}}\,+\,2{{\rm{H}}}^{+}\,\mathop{\longrightarrow }\limits^{{\rm{Au}}}\,2{{\rm{H}}}_{{\rm{2}}}{\rm{O}}\,+\,2{{\rm{h}}}^{+}$$

2 $${\bf{A}}{\bf{n}}{\bf{o}}{\bf{d}}{\bf{e}}:{\rm{Si}}\,+\,6{\rm{HF}}\,+\,4{{\rm{h}}}^{+}\,\to {{\rm{SiF}}}_{6}^{-}\,+\,6{{\rm{H}}}^{+}$$

As described in Fig. 1(a), the holes injected into the underlying Si substrate are mainly consumed by oxidization of Si atoms near the catalytic Au. These oxidized Si atoms are etched by HF in the etching solution. These redox reactions proceed continuously on the Si substrate with the patterned Au catalyst and vertically etched Si nanowires of high aspect ratio are formed by MACE. Although there might be differences depending on the type and concentration of dopants of the Si substrate, holes excessively produced and injected into the Si substrate can be diffused and accumulated in the interior of the substrate or along the nanowires. These excess holes can contribute to additional etchings of the structures and could increase the surface porosity of the nanowires with Si nano-sized grain formation, as described in Fig. 1(b)^2,12,13^.

As mentioned in the introduction, the structures of the MACE-fabricated Si nanowires can be affected by several experimental factors such as temperature, humidity, materials and structures of catalysts, and composition of the etching solution^1,11,14^. The rates of reduction reaction on the Au surface and the hole injection into the Si substrate are critically increased with the oxidant concentration in the MACE process. Surface morphologies and interfacial energy states of the etched Si nanowires are significantly affected by the H_2_O_2_ concentration in the etching solution.

**Figure 2:**
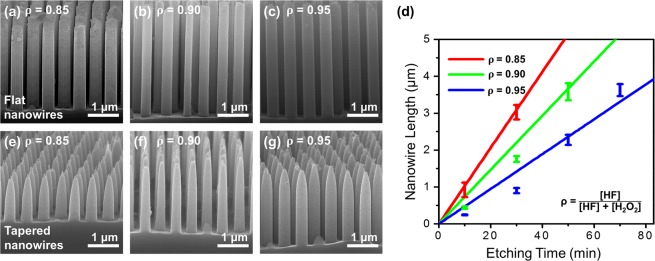
SEM images of vertically etched flat and tapered Si nanowires. Flat nanowires are fabricated in MACE etching solutions of different HF compositions (ρ = [HF]/([HF] + [H_2_O_2_])) of (**a**) 0.85, (**b**) 0.90 and (**c**) 0.95. (**d**) The reaction time is controlled from 30 min to 70 min for each MACE process to obtain Si nanowires with similar lengths of 3 µm. (**e–g**) Flat Si nanowires of different ρ values are anisotropically etched to taper the tip ends of nanowires.

In this study, Si nanowires are fabricated in etching solutions with different HF compositions (ρ, ρ = [HF]/([HF] + [H_2_O_2_]) to investigate the influence of the oxidant on their morphology and structure, as shown in Fig. 2(a–c). The HF concentration is fixed at 3.5 M, while the ρ value is adjusted to 0.85, 0.90, and 0.95 for each etching solutions by controlling the H_2_O_2_ concentration to 0.62, 0.39, and 0.18 M, respectively. All MACE processes are proceeded in etching solutions of different ρ values and the reaction time is changed to control the length of the Si nanowire for each condition, as shown in Fig. 2(d). As the HF composition is increased and the H_2_O_2_ concentration is decreased in the etching solution, the hole-generation and injection into the Si substrate would be decreased during the MACE processes. As a result, the etching rate, determined from the length of the etched nanowires, is decreased to 0.1, 0.08 and 0.06 µm/min with an increase in the ρ value, respectively. In this study, the nanowires are vertically etched to obtain lengths of 3−4 µm for effective light trapping without agglomeration during drying. The reaction times are controlled within 30 to 70 min with ρ values increased from 0.85 to 0.95. Lengths of fabricated nanowires are measured as 3.0 ± 0.3, 3.6 ± 0.2, and 3.6 ± 0.2 µm for each etching conditions. As shown in Fig. 1(b), the ACE processes are performed with these MACE-fabricated Si nanowires to make the nanowires having pencil-like sharpened tip ends for their enhanced light trapping^3,6,22–24^. The ACE process involves reduction of Ag ions (Eq. 3) and oxidation and etching of Si atoms (Eqs. 4 and 5) and these redox reactions are proceeded selectively on reactive Si atoms of sharp edges or Si nano-sized grains of the nanowires^8,18,22^. Ag nanoparticles are grown on the Si nanowire surface during the reduction and completely removed from the Si surface completely in the HNO_3_ solution. These multiple processes are repeated seven times, sequentially to form tapered Si nanowires as shown in Fig. 2(e–g).

3 $${\bf{C}}{\bf{a}}{\bf{t}}{\bf{h}}{\bf{o}}{\bf{d}}{\bf{e}}:{{\rm{Ag}}}^{+}\,+\,{{\rm{e}}}^{-}\to {\rm{Ag}}$$

4 $${\bf{A}}{\bf{n}}{\bf{o}}{\bf{d}}{\bf{e}}:{\rm{Si}}\,+\,{{\rm{2H}}}_{{\rm{2}}}{\rm{O}}\,\to \,{{\rm{SiO}}}_{2}\,+\,{{\rm{4H}}}^{+}\,{+\mathrm{4e}}^{-}$$

5 $${{\rm{SiO}}}_{{\rm{2}}}\,+\,{\rm{6HF}}\,\to \,{{[\mathrm{SiF}}_{6}]}^{2-}\,+\,{{\rm{2H}}}_{2}{\rm{O}}\,+\,{{\rm{2H}}}^{+}$$

Besides changes in the tip ends of the Si nanowires, the lengths of the nanowires are decreased in the ACE process and are measured to be 1.7 ± 0.1, 2.2 ± 0.2, and 2.7 ± 0.1 µm, with an increase in ρ values. Compared the lengths of flat and tapered Si nanowires fabricates with the MACE and ACE processes, tapered nanowires of ACE are shorter than flat nanowires of MACE, showing that length ratios of the tapered nanowires relative to the flat nanowire increase from 0.57 (ρ = 0.85) to 0.73 (ρ = 0.95) with an increase in the ρ value. This dependence can be attributed to that the nanowire surface, etched by MACE of a higher ρ value, i.e., lower H_2_O_2_ concentration, is stable and less oxidized during the MACE process.

**Figure 3:**
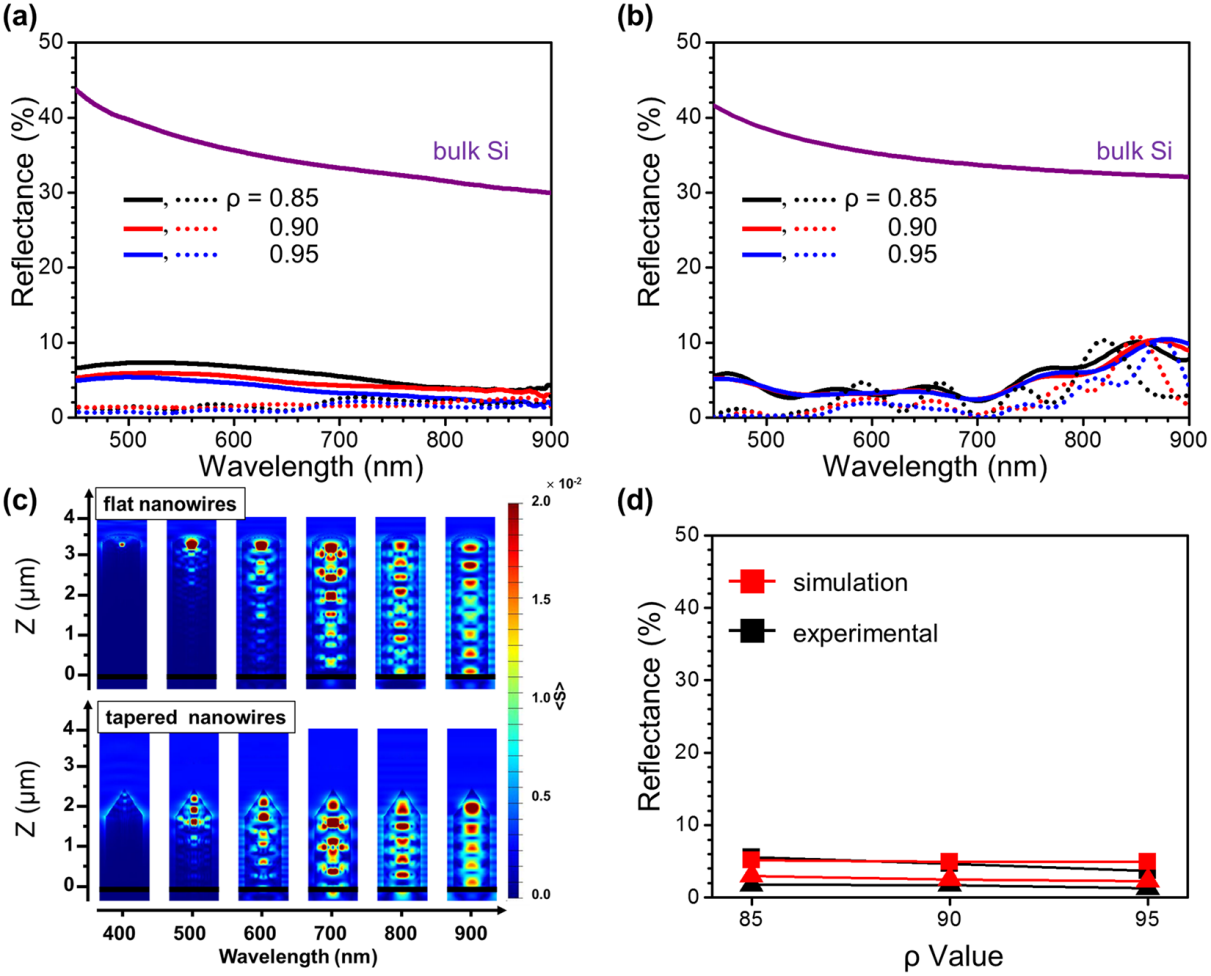
Comparison of (**a**) experimental and (**b**) simulated reflectances of (solid) flat and (dot) tapered Si nanowires of different etching compositions (ρ) of (black) 0.85, (red) 0.90 and (blue) 0.95 and of the (violet) bulk c-Si substrate. (**c**) Cross-sections of distribution of the time-averaged Poynting vector (<S>) near the (upper) flat and (lower) tapered nanowires of ρ = 0.95, calculated in wavelength region of 400–900 nm. (**d**) (black) Experimental and (red) simulated reflectance spectra of flat (■) and tapered (▲) nanowires of each ρ value are averaged in the wavelength range of 450–900 nm for comparison. All of simulations have been performed with a commercialized FDTD solution (Lumerical Solution, Lumerical Inc.)^41^.

As expected, due to anti-reflection enhanced by their sharpened tip structures, the surface reflection of tapered Si nanowires is measured as much lower compared to those of the flat Si nanowires, as shown in Fig. 3(a)^25,26^. Compared to the average reflectance in the wavelength region of 450–900 nm (R_avg_) of 35% of the bulk c-Si substrate, R_avg_ of the flat Si nanowires etched by MACE is significantly decreased to less than 7% for all nanowires. With an increase in ρ values from 0.85 to 0.95, R_avg_ of the flat nanowires decrease from 6.1% to 4.1%. The R_avg_ values of all tapered nanowires fabricated by ACE, further decrease to less than 2% showing a minimum value of 1.1% for the nanowire of ρ = 0.95. Figure 3(b) shows the reflectance spectra of flat (solid lines) and tapered (dots lines) Si nanowires simulated with the FDTD method^3,6,27,28^. The lengths of the Si nanowires considered in the simulation are used according to the SEM images of the nanowires shown in Fig. 2. MACE-fabricated flat nanowires and ACE-fabricated tapered nanowires are assumed to have roughed tip surfaces considering their TEM images shown in Fig. 5 (See the Supporting Information, Figure S1 for detail).

For further understanding of the light trapping of the Si nanowires, we investigate the cross-sections of the time-averaged Poynting vectors, <S>, near the flat and tapered nanowires of ρ of 0.95 at wavelengths ranging from 400 to 900 nm, as shown in Fig. 3(c). <S> represents the power distribution near the structures^29^. Cross-sections of power distributions near the nanowires show significant light trapping of shorter wavelength of <700 nm near the tip of the Si nanowire, indicating that the tip structure of the Si nanowire is critical for its light trapping.

Figure 3(d) shows the averaged values of the simulated (red) and experimental (black) reflectance over the entire wavelength range from 450 to 900 nm of flat (square) and tapered (triangle) Si nanowires with respect to their ρ values. As described above, the significant decrease in the surface reflection, and thus, the enhancement of light trapping of the nanowires through a change of their tip structures is confirmed. The surface reflections of Si nanowires fabricated with MACE and ACE show a slight decrease with an increase in ρ values and this slight change can be explained with increased light trappings of porous nanowires due to the surface structures in their length directions.

**Figure 4:**
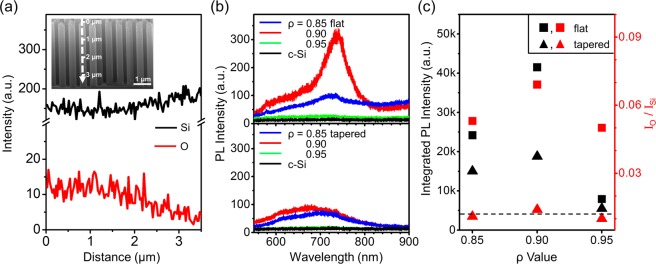
(**a**) EDS line profiles of Si (black) and O (red) components along the flat Si nanowire of ρ = 0.95, indicated as a dash line on the Si nanowire in the SEM image (inset), (**b**) PL spectra of flat (upper) and tapered (lower) Si nanowires of ρ of 0.85 (blue), 0.90 (red), and 0.95 (green) and the PL spectrum of the bulk c-Si substrate (black). (**c**) Comparison of integrated PL intensities of flat (■) and tapered (▲) nanowires at the wavelength range of 550–900 nm and ratios of integrated EDS intensities of O (I_O_) and Si (I_Si_) of flat (■) and tapered (▲) nanowires. The black dash line indicates the integrated PL intensity of the bulk c-Si substrate.

Figure 4(a) shows the EDS line profiles for a comparison of element distributions of O and Si in the longitudinal direction of the Si nanowires, etched by MACE of ρ = 0.95. In these EDS line profiles, the intensity of O was observed more strongly at the tip end of the nanowire than near the substrate^20,30^. The Fig. 4(b) shows a comparison of the photoluminescence (PL) spectra of flat (upper) and tapered (lower) Si nanowires of different ρ values, indicating significant dependence of PL spectra of the nanowires with characteristic PL emissions of porous Si structures near λ = 700 nm on their etching procedures and structures^31–34^. Especially, the flat and tapered nanowires of ρ = 0.95 are observed to exhibit lower PL intensities, similarly to the PL intensity of the bulk c-Si substrate. These EDS line profiles (red) and PL spectra (black) of the flat (■) and tapered (▲) Si nanowires are compared in Fig. 4(c). To compare the O and Si compositions of the flat and tapered Si nanowires fabricated under MACE conditions with different ρ values, EDS line profiles of O and Si were measured from five different nanowires. Ratios of integrated EDS intensities along the nanowires of O (I_O_) and Si (I_Si_) were averaged and compared in the Fig. 4(c). The PL spectra taken from the flat and tapered nanowires are integrated over all wavelength regions, and their integration values are compared in Fig. 4(c) as well.

As described above, hole generations on catalytic Au surfaces during MACE are strongly influenced by H_2_O_2_ concentration in the etching solution and the excess holes injected into the Si substrate are diffused into the substrate and on the nanowires, causing secondary etchings of the structures. In particular, holes diffused into the nanowires are accumulated at their tip end, such that the tip ends become more porous with lots of Si nano-sized grains which can be easily oxidized during and after MACE due to their higher surface energies. Therefore, I_O_/I_Si_ values are observed to be relatively higher on the nanowires etched in etching solutions of lower ρ values, where holes are excessively generated during MACE. Si atoms and grains of higher surface energies can be effectively removed from the nanowires by ACE, showing about ~20% decreases of I_O_/I_Si_ values for the tapered nanowires compared to those of the flat nanowires.

Porous Si nanowires covered with Si nano-sized grains have various energy states available due to the formation of Si/SiO_x_ interfaces and the quantum confinement effect of Si nanocrystals^9,18,20^. Theses defect energy states significantly increase the electron-hole recombination rates and PL emissions of the Si nanowires. Similarly to the EDS analysis, integrations of PL intensities show significant decrease when the nanowires are fabricated in the etching solutions of ρ = 0.95, where the hole injection into the Si substrate and the surface oxidation of the nanowires are minimized. PL intensities of tapered nanowires fabricated with ACE are decreased to 50% as compared to those of the MACE-fabricated flat nanowires. The tapered nanowires of ρ = 0.95 show lowest PL intensities, which are comparable to that of the bulk c-Si substrate (black dash line), indicating that unstable Si atoms and nano-sized grains formed on the Si nanowires are almost removed during the ACE process.

**Figure 5:**
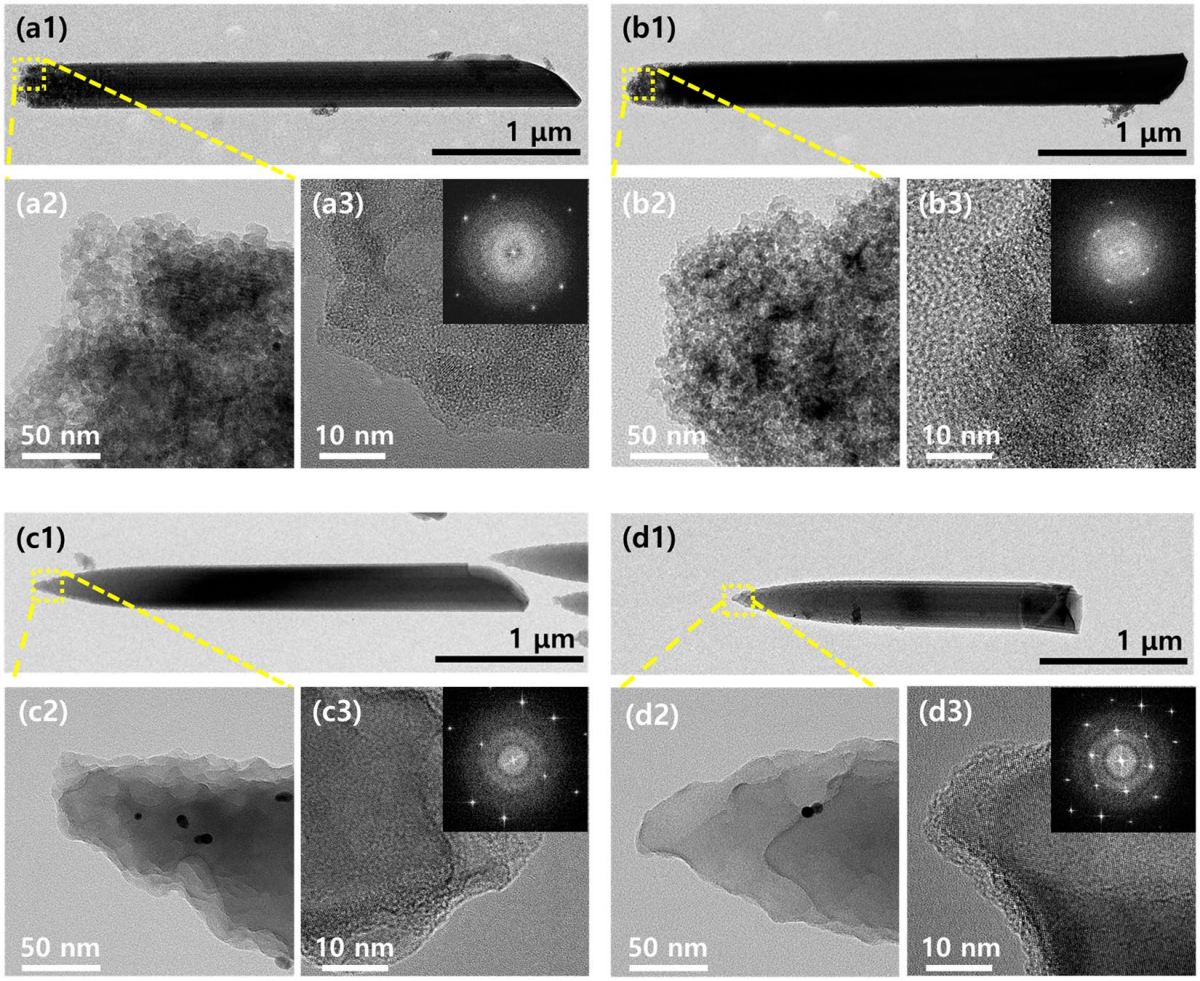
TEM images taken from flat Si nanowires of (a1–a3) ρ = 0.95 and (b1–b3) ρ = 0.90 and from tapered Si nanowires of (c1–c3) ρ = 0.95 and (d1–d3) ρ = 0.90, respectively. Inset figures of HRTEM images of a3, b3, c3 and d3 are diffraction patterns achieved by using the fast Fourier transform (FFT) of the HRTEM images to compare crystallinities of the flat and tapered Si nanowires.

Figure 5 shows transmission electron microscope (TEM) images of flat (a1−a3) and (b1−b3) and tapered (c1−c3) and (d1−d3) Si nanowires of ρ = 0.95 and 0.90, respectively. The Si nanowires are carefully transferred from their substrates onto the TEM grid. TEM images of Fig. 5 (a2) and (b2) show that MACE-fabricated flat Si nanowires have very porous surfaces on their tip ends which are fully covered with lots of Si nano-sized grains, in contrast to middle sides of the nanowires, clearly showing the influence of oxidations occurred during MACE by holes excessively accumulated on tip ends of the nanowires^35–38^. These Si nano-sized grains formed on the nanowire surface can be completely removed during ACE, where Si atoms with higher surface energies could be selectively etched as shown in the TEM images of the tapered nanowires in Fig. 5 (c2−c3) and (d2−d3). The crystalline-structures of the flat and tapered Si nanowires are compared with high-resolution TEM (HRTEM) images taken on the tip ends of each nanowires, as shown in Fig. 5 (a3), (b3), (c3) and (d3). Inset figures show diffraction patterns achieved by using the fast Fourier transform (FFT) of the HRTEM images, indicating that surface of the tapered Si nanowires have higher crystalline qualities than those of the flat Si nanowires.

Raman analysis of fabricated Si nanowires of Figure S2 were performed to support our TEM observations that MACE-fabricated Si nanowires have porous tip surfaces covered with lots of nano-sized Si grains and these grains can be effectively removed from the nanowires by the ACE process. As shown by Figure S2(a), contributions of amorphous Si and SiO_2_, which have characteristic broad Raman peaks below 500 cm^−1^, are negligible in Raman spectra of fabricated Si nanowires compared to contributions of c-Si and Si nanocrystals^33,34^. We assumed that phonon modes of nano-sized Si grains can be described with phonon modes of Si nanocrystals, which have phonon modes at lower energy regions compared to the bulk c-Si (centered at 520.7 cm^−1^) due to their phonon locations^20,38,39^. The size and composition of Si nanocrystals of fabricated Si nanowires of ρ = 0.90 and 0.95 were analyzed with fitting analysis, considering shifts to lower energies and broadenings of Raman peaks as shown in Figure S2(b,c)^20,30^. (see description for Figure S2 in Supporting Information) The comparison in Figure S2 (c) indicates significant decrease in the composition of Si nanocrystals in the nanowires fabricated by the MACE processes with the increase in ρ values and by the ACE processes.

## Conclusions

In summary, we fabricated vertically aligned flat and tapered Si nanowires through chemical wet etchings of the MACE and ACE processes and investigated the influences of oxidants in the MACE etching solutions on surface structures, morphologies and interfacial energy states of fabricated Si nanowires. We showed that the Si nanowire surfaces are significantly affected by the concentration of H_2_O_2_ in the etching solution, which causes excessive injections of holes into the Si nanowires in its higher concentration, increasing the surface porosities of the nanowires with lots of Si nano-sized grains formed on the nanowire surfaces. These porous Si nanowires can be easily oxidized during and after the MACE process and their interfacial energy states are strongly affected by surface structure and oxidation of the nanowires, showing significant changes in the PL spectra with MACE etching conditions. The surface of these porous Si nanowires can be completely cleaned by ACE, which selectively removes unstable Si atoms from the nanowire surface. Flat Si nanowires could be engineered for tapered Si nanowires where Si grains are completely removed from the nanowire surface by the ACE process, showing enhanced light trapping as well as significant suppression of PL intensities. Our study can indicate that the surface oxidation which Si nanowires have during the MACE process can be critical in the surface crystallinities and interfacial energy states of the tapered Si nanowires of the post-MACE etching process. We believe that our study of enhancing surface properties including structural morphologies, crystallinities and interfacial energy states of Si nanowires with the MACE and ACE processes would contribute to the application and fabrication of Si nanowires for efficient Si-based energy harvesting and catalysis.

## Method

### Fabrication of vertically aligned Si nanostructure array

In this study, p-type (boron)-doped Si substrates (100) with the resistivity of 1–10 Ω·cm and thickness of 550 µm were used to fabricate vertically etched Si nanowires through MACE. Si substrates were cut into 1.5 × 1.5 cm^2^ samples and treated with a pure piranha (H_2_SO_4_:H_2_O_2_ = 3:1 v/v) solution and O_2_ plasma to remove impurities remaining on the substrates. Close-packed monolayers of polystyrene (PS) beads of 500 nm diameter (Thermo-Scientific, Inc.) were transferred on precleaned Si substrates by the nanosphere lithography method and the size of PS beads was reduced to 350 nm by the inductively coupled plasma reactive ion etch (ICP-RIE)^40^. Au of 30 nm thickness was deposited on these substrates by using the E-beam evaporator at the deposition rate of 3 Å/s and the substrates were stored in a vacuum desiccator prior to the MACE process.

MACE of the prepared substrates were performed in an etching solution of HF (J. T. Baker, Inc.), H_2_O_2_ (J. T. Baker, Inc.) and deionized water for the fabrication of vertically etched Si nanowires. The concentration of HF was fixed at 3.5 M, the concentration of H_2_O_2_ was changed to control the composition of oxidants and ρ values (ρ = [HF]/([HF] + [H_2_O_2_]) were ranged between 0.85 and 0.95 for the etching solutions. All reactions described in this study were performed in the ice bath of 8 °C to minimize the formation of Si nanocrystals and the surface oxidization of the nanowires during the MACE process. The lengths of the Si nanowires were controlled using the reaction time. Remained PS beads and Au were removed from the substrate by rinsing in chlorobenzene and the diluted *aqua regia* (HCl:HNO_3_ = 3:1 v/v) solution, respectively. The ACE process to taper MACE-fabricated Si nanowires was then performed by repeating processes of reducing AgNO_3_ to Ag nanoparticles on tip ends of the nanowires in the mixed solution of HF and AgNO_3_ and removing reduced Ag nanoparticles from the nanowire surfaces in the diluted HNO_3_ solution. For each cycle of the ACE process, the concentration of HF in the HF and AgNO_3_ solution was fixed to 2.0 M while that of AgNO_3_ was increased by 0.1 mM from 0.4 to 1.0 mM for each cycle^8,22^.

### Characterization

The structures and surface morphologies of vertically etched Si nanowires were characterized during MACE and ACE processes by using the scanning electron microscope (SEM, Hitachi S-4800) and the transmission electron microscope (TEM, Tecnai G2 F30). The Raman and PL spectra of the Si nanowires were measured by using the microscope system (a high resolution Raman/PL spectrophotometer, Horiba LabRAM HR-800) with a 100 × objective (NA = 0.9). The Ar ion laser of λ = 514 nm and ~25 µW was used for PL and Raman studies of the nanowires. The PL spectra of the nanowires were measured in the wavelength region of 550–900 nm and Raman spectra of the nanowires were measured in the wavenumber region of 480–560 cm^−1^. Reflectance of the nanowires were measured in the wavelength region of 450–900 nm.

### Finite-difference time-domain simulation

The simulations were performed using the commercial finite-difference time-domain (FDTD) software package of Lumerical Solution 8.15^41^. The shapes and structures of flat and tapered Si nanowires used in the simulation were estimated from SEM images. All nanowires in the simulations were assumed to have a diameter of 350 nm and a pitch of 500 nm. The lengths of the flat nanowires (the tapered ones) were set as 3.0 (1.7), 3.6 (2.2) and 3.6 (2.7) µm, similarly to the lengths of the nanowires estimated from their SEM images. The reflectance spectra and time-averaged Poynting vector distribution of the nanowires, <S>, were simulated in the range of 400–900 nm. All simulations were performed with perfectly matched boundary conditions on the z-axis and with symmetric and anti-symmetric boundary conditions in the x- and y-directions. Simulated reflectance spectra of MACE-fabricated Si nanowires were treated with the low-pass filter to reduce large fluctuations in spectra which result from overestimated interferences between periodically spaced Si nanowires and coherent simulation light source^42,43^. The refractive index values of n and k of Si taken from the literature were considered in the simulation^44^.

## Supplementary information

Supplementary information
