# Supplementary material for: Enhanced Surface Properties of Light-Trapping Si Nanowires Using Synergetic Effects of Metal-Assisted and Anisotropic Chemical Etchings
Source: Sci Rep. 2019 Nov 4;9:15914. doi: 10.1038/s41598-019-52382-4 (PMC6828753; doi:10.1038/s41598-019-52382-4)
Supplement: Supplementary file 1 — Supplementary information [file 41598_2019_52382_MOESM1_ESM.docx]

**Supporting Information**

**Enhanced Surface Properties of Light-Trapping Si Nanowires Using Synergetic Effects of Metal-Assisted and Anisotropic Chemical Etchings**

Youngsoon Jeong, ^a^ Chanwoo Hong, ^a^ Yeong Hun Jung, ^a^ Rashida Akter, ^a^ Hana Yoon, ^b^ and Ilsun Yoon ^a,*^

^a^ Department of Chemistry, Chungnam National University, Daejeon 34134, Republic of Korea

^b^ Korea Institute of Energy Research (KIER), Daejeon 34129, Republic of Korea

*Corresponding author: Ilsun Yoon (E-mail: [ilsunyoon@cnu.ac.kr](mailto:ilsunyoon@cnu.ac.kr))

*
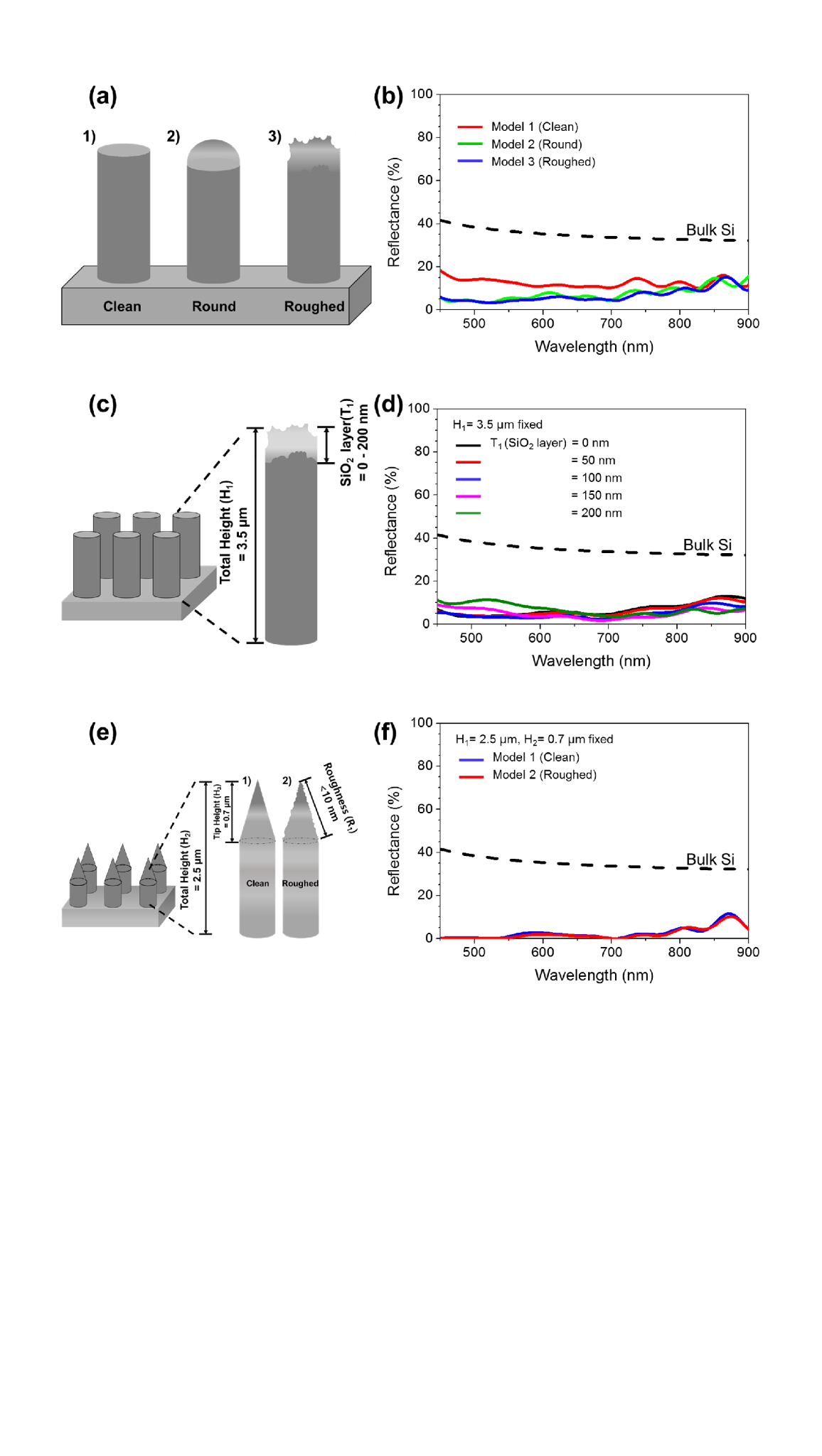
*

**Figure S1.** (a) Tip structures considered for FDTD simulation of MACE-fabricated Si nanowires. (b) Simulated reflectance spectra of Si nanowires of each tip structures: (red) clean, (green) round and (blue) roughed. (c) SiO_2_ layers of 0 to 200 nm are considered to be placed on the roughed tips of Si nanowire. (d) Simulated reflectance spectra of Si nanowires of roughed tip structures with SiO_2_ layers of different thickness. (e) Tapered ACE-fabricated Si nanowires with clean and roughed tip surfaces. (f) Simulated reflectance spectra of tapered Si nanowires of clean and roughed tip surfaces.

In order to consider actual tip structures of MACE-fabricated Si nanowires, FDTD simulations were performed with flat Si nanowires with three different tip surfaces as shown in the schematic in Figures S1(a): clean, round and roughed. The roughed tip surface is assumed to have the maximum profile peak height of up to 50 nm. Averaged reflectances of MACE-fabricated Si nanowires of ρ = 0.95 are decreased from 12.4 % to 7.4 % and 6.6 % as the tip structure is changed from the clean surface to round and roughed surfaces, respectively. These simulation results can indicate reduction in the surface reflection of Si nanowires by structural changes created on tip surfaces during the MACE process.

Additionally, Si nanowires with roughed SiO_2_ layers on the tip surfaces were considered in the FDTD simulations to investigate effects of the reduced effective refractive index due to porous tip surfaces with nano-sized Si grains on the surface reflectance of the fabricated Si nanowires, as shown in Figure S1(c). Averaged reflectances of these Si nanowires are changed from 4.9 % (for the structure with the SiO_2_ layer of 100 nm thickness) and to 6.9 % (for the structure with the SiO_2_ layer of 200 nm thickness), being more comparable to the experimental result (3.7 %) of the MACE-fabricated nanowires. With assumption of the SiO_2_ layer of 100 nm thickness, FDTD simulations were performed for MACE-fabricated flat Si nanowires, as shown in Figure 3(b).

In order to consider effects of the tip surface roughness on the surface reflectance of ACE-fabricated tapered Si nanowires, FDTD simulations were performed with tapered Si nanowires with the clean tip surface and the roughed tip surface, which has the maximum profile peak height of up to 10 nm, as shown in Figure S1(e) and S1(f). As shown by Figure S1(f), the averaged reflectance decreases slightly from 2.6 % to 2.3 % as the roughness of the tip surface increases. The reflectance of the ACE-fabricated Si nanowires shown in Figure 3(b) were simulated with tapered nanowires with roughed tip surfaces.


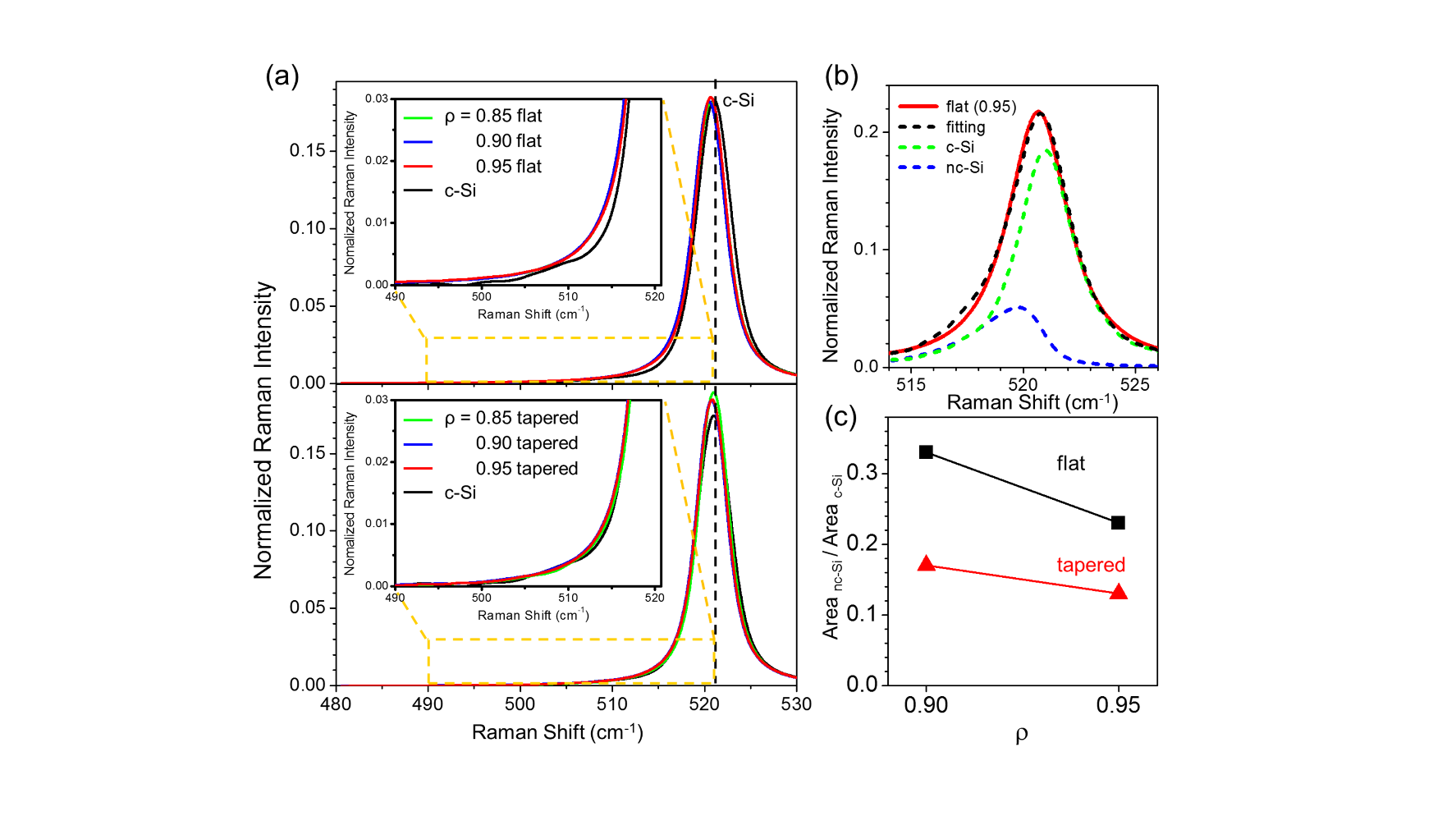


**Figure S2**. (a) Comparison of Raman spectra of (upper) flat and (lower) tapered Si nanowires of different MACE conditions of ρ of (green) 0.85, (blue) 0.90 and (red) 0.95 and of (black) the bulk Si substrate. (b) The normalized Si Raman spectrum of the flat Si nanowire of (red) 0.95 is fitted with the black dash line which includes compositions of (green) bulk c-Si and (blue) Si nanocrystals of size of 9.7 nm. (c) Ratios of integrated area of components of Si nanocrystals relative to those of the (■) flat and (▲) tapered Si nanowires of 0.90 and 0.95.

We performed Raman analysis of fabricated Si nanowires of Figure S2 to support our TEM observations that MACE-fabricated Si nanowires have porous tip surfaces covered with lots of nano-sized Si grains and these grains can be effectively removed from the nanowires by the ACE process.

Figure S2(a) show that contributions of amorphous Si and SiO_2_, which have characteristic broad Raman peaks below 500 cm^-1^, are negligible in Raman spectra of fabricated flat and tapered Si nanowires compared to contributions of c-Si and Si nanocrystals.^1,2^ We assumed that phonon modes of nano-sized Si grains can be described with photon modes of Si nanocrystals, which have phonon modes at lower energy regions compared to the bulk c-Si (centered at 520.7 cm^-1^) due to their phonon locations. The size and composition of Si nanocrystals on flat and tapered Si nanowires can be analyzed with shifts to lower energies and broadenings of Si Raman peaks of the nanowires as shown in Figure S2 (b) and S2 (c).

Assuming that the Si Raman spectra of the nanowires comprise of components of c-Si and Si nanocrystals, the integrated areas of each components in the Raman spectra can be compared to determine the compositions of Si nanocrystals on the nanowires. Here, the size of the Si nanocrystals is determined to be 9.7 nm with a fitting analysis considering the phonon localization of the nanometer-sized Si, similarly to the sizes of Si nanocrystals on the tip ends of the nanowires shown in Figure 5 (a2) and (b2). Compositions of Si nanocrystals (A_Si nanocrystal_ / A_Si nanowires_) in Si nanowires were compared in Figure S2 (c), where A_Si nanowire_ = A_c-Si_ + A_Si nanocrystal_. A_Si nanowire_ is the integrated areas of Raman spectra of the Si nanowires, where A_c-Si_ and A_Si nanocrystal_ are integrated area of Raman spectra of each components in the Si nanowires, respectively.^3,4^

**REFERENCE**

1. Mohammed, M. S. G., Cazzanelli, E., Fasanella, A., Castriota, M. Silicon Nanocrystals on the Surface of Standard Si Wafers: A Micro-Raman Investigation. *Journal of Materials Science and Chemical Engineering* **6**, 104-116. (2018).
2. Theodorakos, I. *et al*. Picosecond and nanosecond laser annealing and simulation of amorphous silicon thin films for solar cell applications. *J. Appl. Phys.* **115**, 013108. (2014).
3. Ratchford, D., Yeom, J. H., Long, J. P. & Pehrsson, P. E. Inﬂuence of inhomogeneous porosity on silicon nanowire Raman enhancement and leaky mode modulated photoluminescence. ***Nanoscale* 7**, 4124-4133. (2015).
4. Dawood, M. K. *et al.* Influence of catalytic gold and silver metal nanoparticles on structural, optical, and vibrational properties of silicon nanowires synthesized by metal-assisted chemical etching. *J*. *Appl. Phys.* **112**, 073509. (2012).
